# ^18^F-FMISO-PET Hypoxia Monitoring for Head-and-Neck Cancer Patients: Radiomics Analyses Predict the Outcome of Chemo-Radiotherapy

**DOI:** 10.3390/cancers13143449

**Published:** 2021-07-09

**Authors:** Montserrat Carles, Tobias Fechter, Anca L. Grosu, Arnd Sörensen, Benedikt Thomann, Raluca G. Stoian, Nicole Wiedenmann, Alexander Rühle, Constantinos Zamboglou, Juri Ruf, Luis Martí-Bonmatí, Dimos Baltas, Michael Mix, Nils H. Nicolay

**Affiliations:** 1 Department of Radiation Oncology, Division of Medical Physics, Medical Center, Faculty of Medicine, University of Freiburg, 79106 Freiburg, Germany; tobias.fechter@uniklinik-freiburg.de (T.F.); benedikt.thomann@uniklinik-freiburg.de (B.T.); dimos.baltas@uniklinik-freiburg.de (D.B.); 2 German Cancer Consortium (DKTK), German Cancer Research Center (DKFZ), Partner Site Freiburg of the German Cancer Research Center (DKFZ), 69120 Heidelberg, Germany; anca.grosu@uniklinik-freiburg.de (A.L.G.); arnd.soerensen@uniklinik-freiburg.de (A.S.); raluca.stoian@uniklinik-freiburg.de (R.G.S.); nicole.wiedenmann@uniklinik-freiburg.de (N.W.); alexander.ruehle@uniklinik-freiburg.de (A.R.); Constantinos.Zamboglou@uniklinik-freiburg.de (C.Z.); juri.ruf@uniklinik-freiburg.de (J.R.); michael.mix@uniklinik-freiburg.de (M.M.); nils.nicolay@uniklinik-freiburg.de (N.H.N.); 3 La Fe Health Research Institute, Biomedical Imaging Research Group (GIBI230-PREBI) and Imaging La Fe node at Distributed Network for Biomedical Imaging (ReDIB) Unique Scientific and Technical Infrastructures (ICTS), 46026 Valencia, Spain; marti_lui@gva.es; 4 Department of Radiation Oncology, Medical Center, Faculty of Medicine, University of Freiburg, 79106 Freiburg, Germany; 5 Department of Nuclear Medicine, Medical Center, Faculty of Medicine, University of Freiburg, 79106 Freiburg, Germany

**Keywords:** hypoxia, ^18^F-FMISO-PET, radiotherapy response monitoring, head-and-neck squamous cell carcinoma and radiomics

## Abstract

**Simple Summary:**

In this study, we monitored FMISO-hypoxia during chemo-radiotherapy (CRT) in head-and-neck cancer patients and we aimed to develop a radiomics model for prediction of treatment outcome. The protocol for the prospective patient cohort (*N* = 35) involved FMISO-PET/CT imaging at three time-points during treatment (weeks 0, 2 and 5). FMISO-hypoxia monitoring was quantified in terms of variations in the size, in the location and in the radiomics features (delta radiomics) of the hypoxia subvolume within the tumor. Local recurrence, distant metastasis, overall survival and progression free survival were employed for the characterization of CRT outcome.

**Abstract:**

Tumor hypoxia is associated with radiation resistance and can be longitudinally monitored by ^18^F-fluoromisonidazole (^18^F-FMISO)-PET/CT. Our study aimed at evaluating radiomics dynamics of ^18^F-FMISO-hypoxia imaging during chemo-radiotherapy (CRT) as predictors for treatment outcome in head-and-neck squamous cell carcinoma (HNSCC) patients. We prospectively recruited 35 HNSCC patients undergoing definitive CRT and longitudinal ^18^F-FMISO-PET/CT scans at weeks 0, 2 and 5 (W0/W2/W5). Patients were classified based on peritherapeutic variations of the hypoxic sub-volume (HSV) size (increasing/stable/decreasing) and location (geographically-static/geographically-dynamic) by a new objective classification parameter (CP) accounting for spatial overlap. Additionally, 130 radiomic features (RF) were extracted from HSV at W0, and their variations during CRT were quantified by relative deviations (∆_RF_). Prediction of treatment outcome was considered statistically relevant after being corrected for multiple testing and confirmed for the two ^18^F-FMISO-PET/CT time-points and for a validation cohort. HSV decreased in 64% of patients at W2 and in 80% at W5. CP distinguished earlier disease progression (geographically-dynamic) from later disease progression (geographically-static) in both time-points and cohorts. The texture feature low grey-level zone emphasis predicted local recurrence with AUC_W2_ = 0.82 and AUC_W5_ = 0.81 in initial cohort (*N* = 25) and AUC_W2_ = 0.79 and AUC_W5_ = 0.80 in validation cohort. Radiomics analysis of ^18^F-FMISO-derived hypoxia dynamics was able to predict outcome of HNSCC patients after CRT.

## 1. Introduction

Radiotherapy constitutes a treatment mainstay for patients with head-and-neck squamous cell carcinomas (HNSCC) and is often combined with concomitant chemotherapy for locally/locoregionally advanced cancers [1,2]. However, the radiation resistance of individual tumors has been shown to strongly depend on the presence and dynamics of tumor-associated hypoxia [3,4,5]. Several means are available to assess and monitor tumor-associated hypoxia, including direct oxygen measurements, gene/protein expression analyses in tissue specimens and hypoxia imaging [6]. In this respect, hypoxia PET/CT allows longitudinal and non-invasive measurement of tumor hypoxia. Additionally, hypoxia PET tracers such as 18F-fluoroazomycin arabinoside (^18^F-FAZA) or 18F-fluoromisonidazole (^18^F-FMISO) have been extensively studied to monitor hypoxia in HNSCC. ^18^F-FMISO-PET has been reported to reflect cell reoxygenation and could therefore be suitable for monitoring therapeutic efficiency [7,8].

Considering the significant influence of tumor-associated hypoxia on the response of HNSCCs to radiotherapy, different treatment strategies have been proposed and tested in exploratory analyses, especially escalating treatment doses to high-risk tumor subvolumes [9,10,11]. Consequently, monitoring spatial and temporal hypoxia responses by PET imaging might provide information to design response-adapted therapy strategies [12]. However, the identification of patients that could benefit from such strategies, as well as the identification of critical subareas is still a matter of discussion, and more studies addressing these issues specifically are needed.

Radiomics is an emerging field developing descriptive or predictive computational models for image analyses to improve individualized diagnosis or treatment selection [13,14,15]. Radiomics relies on the extraction of a large amount of quantitative imaging features (radiomic features) such as first-order statistics (histogram and shape parameters), and second or higher-order statistics simultaneously providing spatial and voxel intensity information (texture features). Radiomics has been successful in the prediction of treatment outcome in several cancer sites: lung [16,17], glioblastoma [18,19] and prostate [20,21]. In our study, we aimed at identifying ^18^F-FMISO radiomics-processed spatial information about critical tumor subvolumes that correlate with adverse outcome and may therefore contribute to models for hypoxia-directed treatment adaptation in HNSCC patients.

## 2. Materials and Methods

### 2.1. Patients

Our study involved 35 patients with histologically confirmed locally advanced HNSCC of the oral cavity, oropharynx, hypopharynx and larynx. All patients underwent definitive CRT. Radiation treatment was delivered as conformal intensity-modulated radiation therapy with a total dose of 70 Gy in 2 Gy fractions: the planning target volume received 50 Gy in 25 fractions and the boost volume additionally received 20 Gy in 10 fractions. The boost target volume was contoured by an expert radiation oncologist, based on clinically relevant regions (both primary tumor and nodal volumes) determined by multimodality imaging. The hypoxic sub-volume (HSV) observed within the boost volume was evaluated. Cisplatin was administered in weeks 1, 4 and 7 (100 mg/m^2^ body surface area). The analysis was applied to two different cohorts: for the initial cohort 25 patients were prospectively recruited between March 2013 and August 2017 and for the validation cohort 10 additional patients were prospectively recruited from January 2019 to October 2020. Clinical characteristics of the patient cohorts are summarized in Table 1. For HPV status definition, P16 overexpression was used as surrogate parameter for tumor’s HPV status, and p16 overexpression was assumed if ≥70% of cells (cytoplasm and nuclei) were p16-positive.

### 2.2. ^18^F-FMISO-PET Imaging

Each patient underwent three ^18^F-FMISO-PET scans: one before CRT (W0) and two additional scans in weeks 2 (W2) and 5 (W5) during CRT. The 10 min ^18^F-FMISO-PET acquisitions were planned 160 min post-injection of a tracer activity of 4 MBq/kg body weight. All patients were imaged with a Philips Gemini TF BigBore 16 PET/CT (Eindhoven, The Netherlands). The scanner fulfilled the requirements indicated in the European Association of Nuclear Medicine (EANM) imaging guidelines and obtained EANM Research Ltd. (EARL) accreditation. PET data were corrected for random- and scatter-coincidences and photon attenuation, based on the corresponding CT dataset. The reconstruction method was a LOR-based ordered-subset iterative time-of-flight algorithm using spherical coordinates (BLOB-OS-TF) with three iterations, 33 subsets and a relaxation parameter for smoothing of 0.35. Of the two isotropic PET reconstruction voxel sizes available in our PET/CT system (4 and 2 mm), the smaller one was chosen (matrix: 512 × 512). This is the PET image resolution currently employed at our institution in the clinical workflow for HNSCC patients. The voxel intensities were normalized to decay-corrected injected activity per kg body weight (standardized-uptake-value SUV (g/mL)).

All patients were immobilized with a thermoplastic head-and-neck mask in the radiation treatment position. ^18^F-FMISO-PET image co-registration was performed using the respective CT images by applying a rigid registration within the open source software 3D-Slicer (https://www.slicer.org/, accessed on 9 June 2021). Registered images were visually confirmed or corrected before being involved in the analysis.

### 2.3. Segmentation

Based on previous publications, HSV was determined by using a target-to-background-ratio (TBR) of 1.4 [22,23]. A detailed description of background (BG) segmentation is provided in the Appendix A. The proposed semi-automatic segmentation method minimizes inter- and intra-variability. HSV were segmented on each of the three ^18^F-FMISO scans: HSV_W0, HSV_W2 and HSV_W5.

### 2.4. Parameters for the Quantification of Variations in Size and Localization of Hypoxia Volumes

Changes in size of hypoxia during treatment (increasing-hypoxia (IH), stable-hypoxia (SH) and decreasing-hypoxia (DH)) and changes in localization (geographically-static (gS) and geographically-dynamic (gD)) were evaluated [24,25]. For this purpose, a new classification parameter (CP) was proposed to objectively quantify the variation of hypoxia. This CP used the relative volume difference (∆V) and three parameters accounting for spatial overlapping, namely dice-similarity-coefficient (DICE), sensitivity (Sens.) and positive-predictive-volume (PPV). In this way, the high subjectivity conveyed by a visual classification assessment was avoided. A detailed description of the parameters accounting for spatial overlapping is provided in the Appendix B.

**Figure 1:**
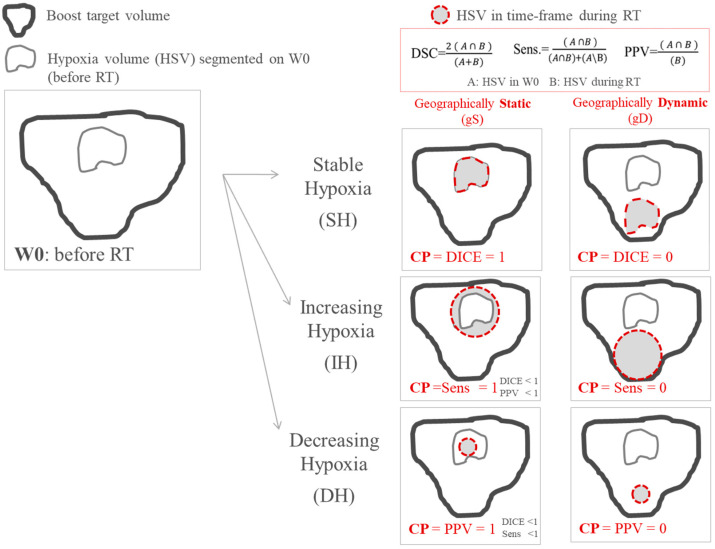
Scheme of the classification employed for classifying variations in size and location of tumor hypoxia during treatment. The classification parameter (CP) takes into account variations in size and location simultaneously.

The CP takes into account changes of the HSV size based on relative volume difference: increasing-hypoxia when the volume increases more than 15% (IH: ∆V ≥ 15%), stable-hypoxia when hypoxia does not vary more than 15% (SH: 15% > ∆V > −15%), and decreased-hypoxia when volume decreases more than 15% (DH: ∆V < −15%). The 15% criterion for stability is based on previous investigations [26]. Once ∆V has been calculated, depending on the resulted classification (IH, SH or DH), CP is defined by different parameters: for stable hypoxia CP = DICE, for increasing-hypoxia CP = Sens. and for decreasing-hypoxia CP = PPV. In this way, geographically-static hypoxia (total overlapping of the contours) corresponds to CP = 1 independently of the variation in size of the hypoxia volumes. By using the combination of the 3 parameters (DICE, Sens. and PPV) in CP definition, we could therefore distinguish between static (high values of CP) and dynamic hypoxia (low values of CP) independently of the variation in hypoxia size, Figure 1.

### 2.5. Radiomics Features Extraction

The segmentation of hypoxia on the ^18^F-FMISO-PET image acquired before treatment (HSV_W0) was transferred to all co-registered PET series. The 130 radiomic features [27] were computed for each ^18^F-FMISO time-point using an open-source code based on MATLAB^®^ (The MathWorks Inc., Natick, MA, USA) [28]. As recommended by previous investigations, SUV values of the voxels within the contour were discretized with a fixed bin width (W = 0.01) for texture feature computation [27,29]. Texture features were derived from five matrices: the 3D version of the gray-level co-occurrence matrix (GLCM); the gray-level run length matrix (GLRLM), the gray-level size zone matrix (GLSZM) and the neighborhood gray tone difference matrix (NGTDM). A wavelet band-pass filtering (WF), with a weight ratio 1:2 between band-pass sub-bands and other sub-bands, and an equal-probability quantization algorithm (Q), by using the function histeq of MATLAB^®^, were also obtained. The features used in this study are listed in the supplementary material (Table S1).

Relative differences in radiomic features (∆_RF_) between W0 and W2 and between W0 and W5 were calculated. The correlations between the 130 ∆RF and the treatment outcome were evaluated.

### 2.6. Statistical Analysis

Statistical analysis was performed using an in-house software based on Wolfram Mathematica v 11.2 (Wolfram Research Europe Ltd., Witney, UK).

The Fisher-ratio-test was applied for the comparison of clinical characteristics in the training and the validation cohort. In the analysis of treatment response, overall survival, progression-free survival, locoregional recurrence and distant metastases were evaluated. For overall and progression-free survival, time intervals were calculated from the start of the treatment. Kaplan–Meier curves were estimated, and groups were compared with the log-rank test. Multivariate Cox regression was used for estimation of hazard ratios (HR) with 95% confidence intervals (CI). In the analysis of binary outputs (local recurrence and distant metastases), the Mann–Whitney U test was used for non-pairwise comparison of groups. An open-source code for binary logistic regression analysis was applied [28]. It involved imbalance-adjusted bootstrap resampling in prediction performance estimation and in the computation of model coefficients. In order to avoid redundancy, we also assessed strong correlations (Spearman test with *r* > 0.8) between different ∆_RF_ showing good prediction performance, and only the feature with the most statistically significant prediction was selected. *p*-Values were adjusted for multiple testing by controlling the false discovery rate with Benjamin and Hochberg’s method [30].

Outcome prediction analyses were done for the relative deviation observed at W2 and afterwards validated regarding the relative deviations for W5. The prediction value of ∆_RF_ was only considered relevant for positive findings at both time-points. In addition, a second patient cohort was used as a validation cohort in order to confirm the results.

## 3. Results

### 3.1. Variations in Size and Localization of Hypoxia Volumes: CP Predicts Progression Free Survival

**Figure 2:**
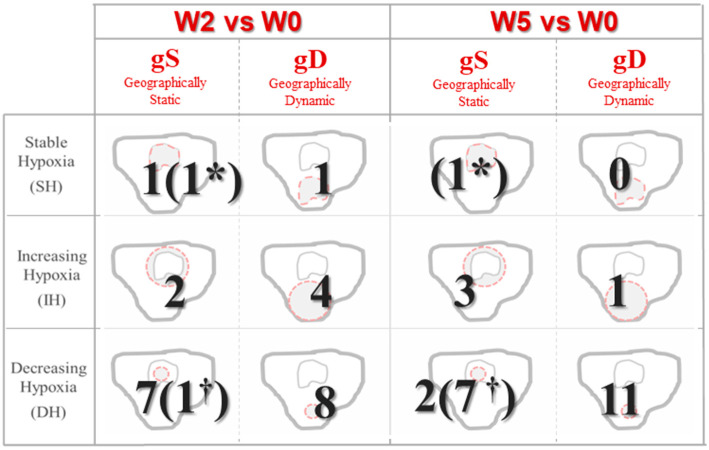
Status classification based on variations during treatment in size and localization of hypoxic volumes. Patients without hypoxic volume in the current time-frame by † and patients without hypoxic volume at both W0 and the current time-frame are represented by *.

Inhomogeneous dynamics of HSV over time were observed for the analyzed patients. The number of patients that could be classified in each hypoxia variation status according to ∆V and CP is presented in Figure 2. Decreasing hypoxia volumes were observed in 16 of 25 patients (64%) at W2, and in 20 patients (80%) at W5. Only 24% of patients showed geographically-stable tumor hypoxia (CP>>) during the whole treatment. Detailed information of hypoxia variation status and treatment outcome for each patient and each week is provided in the supplementary materials, Figure S1.

**Figure 3:**
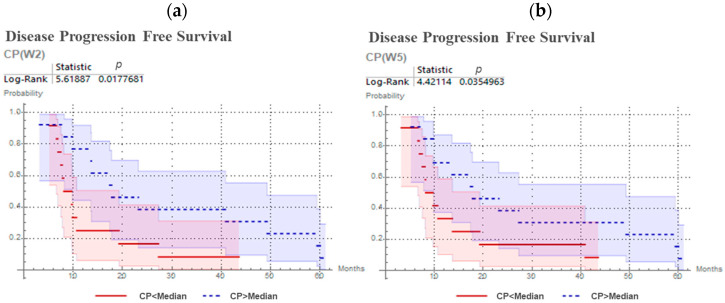
Kaplan–Meier curves and log-rank test for CP predicting progression free survival for head and neck cancer patients, with 18F-FMISO PET/CT at second week (**a**) and at fifth week (**b**) of chemoradiotherapy.

The predictive values for CP, ∆V, DICE, PPV and Sens. were investigated, and results are presented in Table 2. CP distinguished between patients with earlier and later progression at W2 (*p* = 0.018) and its prediction was confirmed at W5 (*p* = 0.035). No other parameter (∆V, DICE, PPV, Sen.) has shown statistically significant outcome prediction independently of the time-point of the ^18^F-FMISO acquisition. Kaplan–Meier curves for CP are presented in Figure 3. Specifically, patients with higher values of CP (geographically-static HSV), exhibited significantly prolonged progression free survival.

### 3.2. Variations in Radiomic Features: Prediction of Local Recurrence and Progression Free Survival

**Figure 4:**
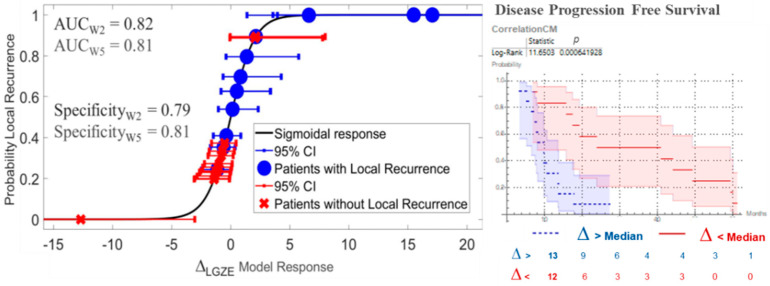
Prediction of treatment outcome by variations in low gray-level zone emphasis (∆_LGZE_) and by variations in the correlation of co-occurrence matrix (∆C_CM_). On the left, the model for prediction of local recurrence by ∆_LGZE_ at W5 is shown. On the right, Kaplan–Meier curves and log-rank test for prediction of disease progression free survival by ∆C_CM_ at W2 are shown. CI = Confidence Interval.

**Figure 5:**
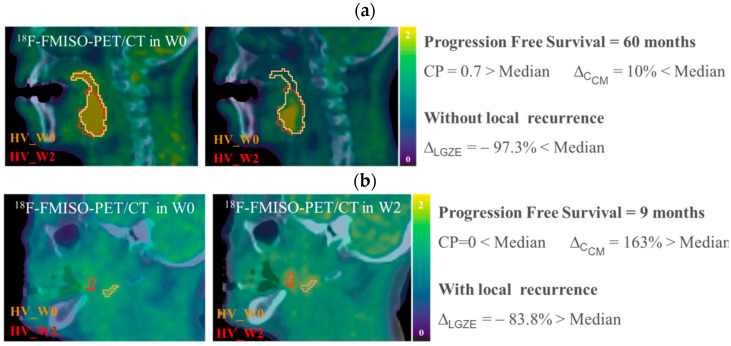
Example of ^18^F-FMISO-PET/CT images, HSV contours and values of CP, ∆_C_CM__ and ∆_LGZE_, for (**a**) P01, patient without local recurrence and longer progression free survival, and for (**b**) P20 patient with local recurrence and shorter progression free survival. All ^18^F-FMISO-PET images were represented with a scale of (0, 2) g/mL and superimposed to the corresponding CT.

From the 130 radiomic features analyzed in this dataset, 35 were able to predict treatment outcome at W2. They are listed in Table S2, supplementary material. For 18 of them, the outcome prediction was confirmed at W5, see Table 3. The 18 radiomics features with predictive value are listed in Appendix C. No one of the clinical parameters in Table 1 showed statistically significant outcome prediction, when applying Benjamin–Hochberg correction for multiple testing. After removing redundant radiomics features by Spearman’s correlation test, the best prediction performances were observed for variations in low gray-level zone emphasis (LGZE) and for variations in the correlation of co-occurrence matrix (C_CM_), Figure 4. Concretely, the relative deviation of LGZE (∆_LGZE_) discriminated between patients with and without locoregional recurrence, with an area-under-the-curve (AUC) of 0.82 and a specificity of 0.79 at W2 and AUC of 0.81 and specificity of 0.81 at W5. In addition, ∆_C_CM__ distinguished between patients with earlier and later progression with *p* = 0.003 at W2 and *p* < 0.001 at W5. Examples for two patients are shown in Figure 5.

### 3.3. Confirmation of the Abbility to Predict Treatment Outcome by CP and ∆_LGZE_ in the Validation Cohort

In order to validate the outcome predictions performance observed for CP, ∆_LGZE_, and ∆_C_CM__ in a second patient cohort, we prospectively recruited 10 additional patients. Clinical characteristics of the validation cohort are presented in Table 1. Local recurrence was observed for only one patient. Consequently, for the part of the validation focused on the prediction of the local recurrence by ∆_LGZE_, 5 patients with local recurrence from the initial cohort were included.

**Figure 6:**
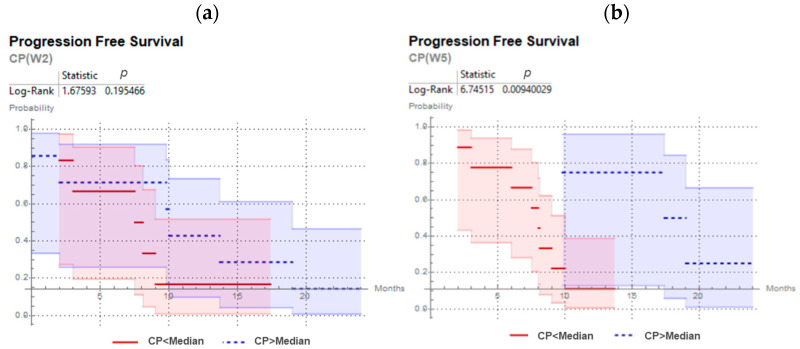
Kaplan–Meier curves and log-rank test for CP predicting progression free survival for head and neck cancer patients of the validation cohort, with ^18^F-FMISO PET/CT at second week (**a**) and at fifth week (**b**) of chemoradiotherapy.

Results are presented in Table 4. The prediction of disease progression by *∆_CCM_* was not confirmed in the validation cohort. ∆_LGZE_ predicted local recurrence with very good performance independently of the time-point. In addition, disease progression prediction by CP was statistically significant (*p* = 0.009) at W5. At W2, although the disease progression prediction by CP was not statistically significant (*p* = 0.19), patients with geographically-stable hypoxia (CP>>) showed an average for disease progression of 11.21 months and patients with dynamic hypoxia (CP<<) showed earlier progression with an average of 7.8 months, Figure 6.

## 4. Discussion

To the best of our knowledge, we presented the first study that simultaneously linked variation in size, location and heterogeneity of hypoxia distribution based on ^18^F-FMISO-PET image quantification to the outcome of HNSCC patients undergoing CRT. We proposed a new classification parameter to objectively quantify temporary variations in hypoxia localization. This classifier showed a statistically significant prediction of treatment outcomes in our dataset. Additionally, the application of radiomics analysis on longitudinal ^18^F-FMISO images showed that the texture feature low grey-level zone emphasis predicted local recurrence. Results for the CRT-outcome prediction were successfully confirmed on a second ^18^F-FMISO acquisition time-point during treatment (W5) and in a validation cohort.

It was observed that the percentages of patients showing decreasing hypoxia during the course of treatment (64% in W2 and 80% in W5) were in good agreement with the remarkable reduction in tumor hypoxia observed in most studies performed so far. Specifically, in previous publications, decreasing or resolving hypoxia during or after treatment was more frequent than residual or increasing hypoxia with percentages of 89% in W4 [27], 58% in W5 [19], 50% in W4 [5] and 79% in W4–W5 [28]. In our cohort, although a trend towards decreasing hypoxia volumes during treatment was confirmed, no statistically significant correlation between variations in size (∆V) and treatment outcome could be observed. However, the variations in hypoxia localization quantified by CP (static against dynamic) were able to significantly predict treatment outcomes independently of the acquisition time of the ^18^F-FMISO-PET/CT exam. Patients with hypoxia remaining in the same location during treatment had later disease progression (recurrence, metastasis or death) than patients with dynamic hypoxia. Therefore, measuring spatial hypoxia dynamics during CRT seems useful to monitor treatment and to develop treatment adaptation strategies for HNSCC patients.

In HNSCC, previous publications have shown significant correlations between tumor hypoxia and metastasis [29] or between hypoxia and decreased OS [28,30,31,32,33,34]. In our study, in addition to the outcome prediction by CP discussed above, relative deviations for the texture feature ∆_LGZE_ were able to differentiate between patients with and without local recurrence. LGZE is a texture feature that quantifies tracer (^18^F-FMISO) distribution heterogeneity by emphasizing regions with low concentration. In our initial cohort, LGZE decreased in both time-points: µLGZE(W0) = 0.05 and µLGZE(W2) = µLGZE(W2) = 0.01. As reference for LGZE values, the NEMA-phantom sphere (diameter = 37 mm) in the EARL-Accreditation measurement presented a value for LGZE (homogeneous) of 0.01 [35] and for a set of heterogeneous home-made simulated lesions LGZE (heterogeneous) values were around 0.0001 [36]. From the correlation between LGZE and local recurrence observed at both time-points, we could conclude that local recurrence is less probable for patients in which ^18^F-FMISO heterogeneity within the initial hypoxic volume increased during treatment. The lower probability of local recurrence associated with an increase in the ^18^F-FMISO heterogeneity (increasing low concentration regions) might be explained by an improvement in tumor cell re-oxygenation given by CRT. This behavior was confirmed in the validation cohort. Local recurrence prediction was statistically significant independently of the ^18^F-FMISO-PET acquisition time-point during CRT. This result implies an important advantage for the implementation of ^18^F-FMISO radiomics monitoring in clinical routine and may provide a way of rendering resource-intensive longitudinal measurements obsolete. Furthermore, this prediction model would allow to overcome the variability in tumor hypoxia quantification by ^18^F-FMISO imaging [37,38,39] and to avoid determining the optimal ^18^F-FMISO-PET time point during treatment [19,20].

Our findings may also have clinical implications for the radiation therapy of HNSCC patients. Dynamic hypoxia within the tumor volume may confer a worse prognosis and may therefore require treatment modification in these patients. However, escalation of the treatment doses, as proposed, may not adequately target areas of dynamic hypoxia and, hence, other means such as systemic hypoxia modification (e.g., with nimorazole) may be more advisable in this setting [40,41,42]. Additionally, the radiomic feature LGZE may predict significant hypoxia reduction over the course of treatment and in this respect help to identify beneficial ^18^F-FMISO dynamics early into treatment. Considering that early information of dynamic tumor hypoxia is crucial to dynamically adapt and personalize treatment strategies, the radiomics data may hold potential stratifying HNSCC treatment as they proved independent of the ^18^F-FMISO scanning time point.

Methods involved in our analysis (segmentation, objective parameter for quantification of variations in size, computation of image features based on open-source code, validation tests conventionally performed (KM, log-rank) or open-source, etc.) have been chosen with the purpose of being easily reproducible by other institutions. Concerning LGZE, in our previous evaluation with experimental phantom measurements [43] LGZE has been proved to be robust with respect to: 3 different PET/CT systems, CT metal artifacts in a head-and-neck configuration and two PET segmentation approaches (40% threshold and a tumor-to-background based threshold). From all these findings the predictive value of LGZE in a new cohort of patients from other institutions could be expected.

Cervical nodal necrosis is a negative prognostic parameter for HNSCC patients undergoing radiotherapy [44,45]. As FMISO has been shown to accumulate in viable but not in necrotic cells, FMISO does not accumulate in the necrotic core but rather in the perinecrotic hypoxic region [46]. In malignant brain tumors, there was a strong correlation between the FMISO uptake and the presence of histopathological necrosis [47]. As we did not routinely perform post-therapeutic tissue biopsies or post-therapeutic multiparametric MRI, we were not able to quantify tumor necrosis after chemoradiation in our cohort. It is known that tumor cells can become apoptotic or necrotic in case of long-lasting severe hypoxia [48]. Geographically static hypoxia over several weeks may therefore result in necrosis induction. Warren and Partridge have incorporated those considerations and developed a computational model of FMISO uptake [49]. In this context, it remains to be elucidated whether the development of tumor necrosis could explain the different prognostic role of geographically static and non-static hypoxia.

The main limitation of this study is the relatively small size of the clinical data sample. For a proper understanding of the sample size, the complexity implied by the recruitment of patients for this prospective trial should be taken into consideration: immobilized patients were imaged by different modalities (FGD-PET, FMISO-PET, CT and MR), acquisitions had to be performed at three different time-points during treatment (W0, W2 and W5) and by the same system. While the size of our cohort was limited (*N* = 35), the number of patients is still comparable or larger than the cohort size evaluated in previous related publications [5,19,28]. The size of our data samples prevented us from reporting a prediction model and from following an optimal workflow for a proper model validation [50]. Concretely, in the prediction of progression free survival by CP, the analysis focused on the quantification of the discrimination in terms of differences (log-rank test) of Kaplan–Meier curves for each subgroup (CP high, CP low). However, no model was reported. If our results are confirmed in a larger data sample, a model for prediction of progression free survival based on CP values will be reported. The accuracy of the model should then be quantified by Harrell’s C-index [51], which is a robust estimator of the concordance between outcome and prediction of the model. In addition, for the validation of the prediction of local recurrence by LGZE, patients from the initial cohort were included. This was necessary in order to have a representative sample of local recurrence (40%), even though it compromises the significance of the accuracy performed by the prediction. Nevertheless, we should remark that a selective analysis was employed in order to ensure the significance of the predictions reported and in order to reject ^18^F-FMISO quantification parameters casually linked with treatment outcome. Concretely, on the one hand, our criteria employed correction for multiple testing to identify statistically significant correlations. On the other hand, outcome predictions by imaging features were validated for a second ^18^F-FMISO acquisition time-point and a validation cohort. Although a larger data sample is required in order to confirm our results, the current study already provides an internal validation, and the findings reported here should be understood as a proof-of-concept to support further investigations of the role of hypoxia for the chemoradiation treatment of HNSCC patients.

## 5. Conclusions

From our knowledge, this is the first study assessing variation in size, location and heterogeneity of hypoxia distribution based on longitudinal ^18^F-FMISO-PET quantification during CRT of HNSCC patients and confirms a trend of decreasing-hypoxia during CRT. In our data sample, variations of volume and target-to-background of HSV did not show statistically significant prediction of the treatment outcome; however, the CP, which quantifies variation in location of HSV, and the ∆_LGZE_, which quantifies the variations in the number of low ^18^F-FMISO concentration regions, showed predictive value and could therefore be of interest for the definition of adaptive radiation therapies. The proposed CP may help to facilitate the classification and comparison of hypoxia variations, and to predict patient outcomes. The predictive value of the texture feature LGZE additionally supports the role of hypoxia monitoring in radiation oncology.

## Figures and Tables

**Figure A2:**
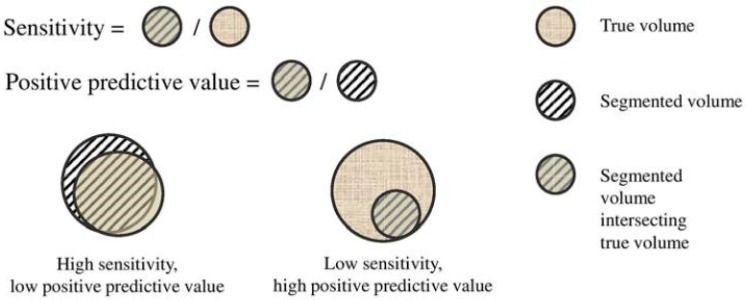
Graphical ilustration of sensitivity (Sen.) and positive predictive value (PPV).

**Table 1:** Clinical characteristics of the patient cohorts.

Clinical Characteristics	Initial Cohort (*n* = 25)	Validation Cohort (*n* = 10)
Clinical Trial Register	DRKS00003830	DRKS00003830
Age (years, mean ± standard deviation, range)	(59 ± 8, 41–79)	(63 ± 9, 48–76)
Sex Female/Male	2 (8%)/23 (92%)	2 (20%)/8 (80%)
Smoker (yes)	20 (80%)	9 (90%)
HPV-Status (positive)	6 (24%)	4 (40%)
T-Stage (*) T2/T3/T4	1 (4%)/8 (32%)/16 (64%)	1 (10%)/5 (50%)/4 (40%)
T-Site oral cavity/oropharynx/hypopharynx/larynx/	2 (8%)/9 (36%)/8 (32%)/1 (4%)/5 (20%)	1 (10%)/6 (60%)/1 (10%)/0 (0%)/2 (20%)
N-Stage N0/N1/N2b/N2c/N3	2 (8%)/1 (4%)/5 (20%)/17 (68%)/0 (0%)	0 (0%)/2 (20%)/2 (20%)/5 (50%)/1 (10%)
Local Recurrence (yes) *	12 (48%)	1 (10%)
Distant Metastasis (yes)	6 (24%)	3 (30%)
Progression Free Survival * (months, mean ± standard deviation, range)	(22 ± 19, 3–61)	(8 ± 7, 0–24)
Overall Survival * (months, mean ± standard deviation, range)	(32 ± 16, 5–61)	(11 ± 8, 0–24)

(*) *p* < 0.05 clinical characteristics not comparable between cohorts.

**Table 2:** *p-*Values for the prediction of the treatment response at week 2 and 5 (W2/W5) during CRT. No statistically significant prediction was represented by “n.s.”.

Parameters	Overall Survival W2/W5	Progression Free Survival W2/W5	Local Recurrence W2/W5	Distant Metastasis W2/W5
Relative volume difference (∆V)	n.s./n.s.	n.s./n.s.	n.s./0.026	n.s./n.s.
Dice Similarity Coefficient (DICE)	0.049/n.s.	0.004/n.s.	0.004/n.s.	0.018/n.s.
Positive Predictive Value (PPV)	n.s./n.s.	n.s./0.046	n.s./0.046	n.s./n.s.
Sensitivity (Sens.)	n.s./n.s.	n.s./n.s.	n.s./n.s.	n.s./n.s.
Classification Parameter (CP)	n.s./n.s.	0.018/0.035	n.s./0.041	n.s./0.043

**Table 3:** Number of FMISO radiomic features able to predict treatment outcome. Benjamin–Hochberg multiple-test correction was applied to determine statistical significance.

Time Point	Overall Survival	Local Recurrence	Distant Metastasis	Progression Free Survival
W2	6	17	2	10
confirmed at W5	0	15	0	3

**Table 4:** Prediction of treatment outcome in the validation cohort: progression free survival by CP progression free survival by ∆_CCM_ and local recurrence by ∆_LGZE_.

Time-Point	CP	∆_CCM_	∆_LGZE_
W2	*p* = 0.19	*p* = 0.49	AUC = 0.78 specificity = 0.89
W5	*p* = 0.009	*p* = 0.29	AUC = 0.79 specificity = 0.76

**Figure A1:**
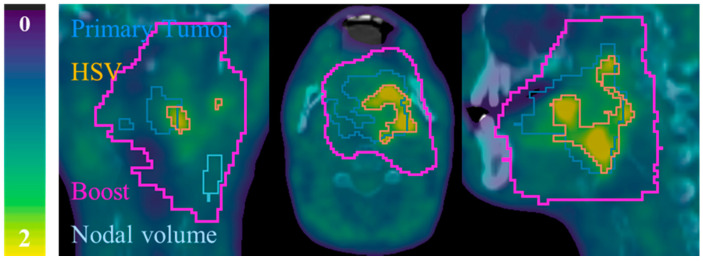
Example of contours for patient P08. ^18^F-FMISO-PET/CT slices: coronal (left), axial (middle) and sagittal (right). All ^18^F-FMISO -PET images of were represented with a scale (0, 2) g/mL and superimposed to the corresponding CT.

## Data Availability

No new data were created or analyzed in this study. Data sharing is not applicable to this article.

## Supplementary Materials

The following are available online at https://www.mdpi.com/article/10.3390/cancers13143449/s1, Table S1: Radiomics Features, Table S2: Radiomics features with prognostic value at W2 in the initial cohort, Figure S1: Hypoxia variation status and treatment outcome for each patient and in each week.

## Appendix A. Automatic FMISO Segmentation Using the Parotid Contour from Radiotherapy Planning

Segmentation of FMISO hypoxia is usually based on a threshold of 1.4 to 1.8 times the mean standard-uptake value (SUV_mean_) within a manual contour of muscle [22]. In order to facilitate the implementation of hypoxia volumes in RT and to minimize the variability observed for manual delineation, we proposed a simple and automatic FMISO segmentation of the background, using the parotid contour from radiotherapy planning. Segmentation process involved the following three steps:

- Firstly, CT from radiotherapy planning is co-registered with FMISO-image by the corresponding CT and contralateral parotid contour (V_parotid_) is transferred to all FMISOs.
- Then, for each voxel of V_parotid_ the intensities derived from the tree FMISOs (W0, W2 and W5) were multiplied and voxels that resulted in zero were removed from the contour. This step took into account the possibility of having part of the V_parotid_ out of the FMISO image.
- Finally, the background (Bg_parotid_) was defined selecting the voxels with highest uptake until filling a volume of 6 mL. By focusing on higher uptake voxels, we could minimized the partial volume effect (PVE) given in voxels in the peripheral zone of the body and the co-registration error. Additionally, a volume of 6 mL permitted to minimize the PVE in the computation of SUV_mean_.

Our proposed method for background definition was compared with the manual delineation of the muscle employed as background in previous publications [23]. Therefore, a sphere within the sternocleidomastoid was manually delineated by an oncologist and established as a reference for background (Bg_ref_). SUV_mean_ stability during the treatment and comparison of SUV_mean_ for both background approaches (Bg_ref_ and Bg_parotid_) were evaluated by Wilcoxon-Rank-Test (WRT) and Bland-Altman-Plot-analysis (BA). Results proved that Bg_ref_ and Bg_parotid_ provided comparable SUV_mean_. In addition, the stability of SUV_mean_ during RT was also confirmed for both contours, with non-statistically significant variations. Consequently, results confirmed that the contour of parotid, required for RT planning, could be employed for automatic FMISO segmentation, avoiding an additional manual delineation of the muscle. The method proposed could therefore minimize the time and the variability implied by manual contouring and consequently, in the current paper BG refers to Bg_parotid_.

The HSV was therefore determined by applying a threshold of 1.4 times SUV_mean_ (BG). All voxels within the boost target volume of the radiotherapy planning with SUV values above this threshold were segmented. Only the largest closed surface was labelled as hypoxia. See example in Figure A1. We could validate our approach by visual inspection: we did not observe hypoxia within the boost incorrectly rejected. The boost target volume was contoured by an expert radiation oncologist, based on clinically relevant regions (both primary tumor and nodal volumes) determined with multimodality imaging. In the boost volume a total dose of 70 Gy was delivered.

## Appendix B. Parameters Accounting for Spatial Overlapping

For a reference segmented volume (A) and a second segmented volume (B), the parameters accounting for spatial overlapping are defined as follows:

- Dice Similarity Coefficient (DSC) which represents the size of the union of two volumes [52]:
  (A1) $$\mathrm{DSC}=\frac{2\left(A\cap B\right)}{\left(A+B\right)}$$
  (0: no spatial overlap, 1: complete overlap)

- Sensitivity (Sen.) and Positive Predictive Value (PPV). To take into account the false positives (FP: Error Type I) and false negatives (FN: Error Type II), Sen. and PPV are employed, Figure A2:
  (A2) $$\mathrm{Sen}=\frac{\left(A\cap B\right)}{\left(A\cap B\right)+\left(A\backslash B\right)}=\frac{\mathrm{TP}}{\mathrm{TP}+\mathrm{FN}}$$
  (A3) $$\mathrm{PPV}=\frac{\left(A\cap B\right)}{\left(B\right)}=\frac{\mathrm{TP}}{\mathrm{TP}+\mathrm{FP}}$$
  where TP means true positives.

## Appendix C. Radiomic Features with Predictive Value Independentl of the Time-Point during Treatment

Local recurrence: LGZE, SZLGE, LZLGE, LGRE, SRLGE, LRLGE, WFLGZE, WFSZLGE, WFLZHGE, WFLGRE, WFSRLGE, WFLRLGE, WFGLV2, QLGZE and QSZLGE.

Progression free survival: C_CM_, LZLGE, WF-Busyness.

All image features are defined in Table S1 of the supplementary materials.
