# Supplementary material for: 18F-FMISO-PET Hypoxia Monitoring for Head-and-Neck Cancer Patients: Radiomics Analyses Predict the Outcome of Chemo-Radiotherapy
Source: Cancers (Basel). 2021 Jul 9;13(14):3449. doi: 10.3390/cancers13143449 (PMC8303992; doi:10.3390/cancers13143449)
Supplement: Supplementary file 1 [file cancers-13-03449-s001.zip › cancers-1273679 supplementary.pdf]

cancers

MDPI

Supplementary Materials

^{18}F-FMISO-PET Hypoxia Monitoring for Head-and-Neck Cancer Patients: Radiomics Analyses Predict the Outcome of Chemo-Radiotherapy

Montserrat Carles, Tobias Fechter, Anca L. Grosu, Arnd Soerensen, Benedikt Thomann, Raluca G. Stoian, Nicole Wiedenmann, Alexander Rühle, Constantinos Zamboglou, Juri Ruf, Luis Martí-Bonmatí, Dimos Baltas, Michael Mix and Nils H. Nicolay

Table S1. Radiomics Features.

SUV-Histogram	Maximum SUV (SUV_{max}), Peak SUV (SUV_{peak}), Minimum SUV (SUV_{max})<br>Mean SUV (SUV_{mean}), Volume (V), Total Lesion ^{18}F-FMISO (TLF),<br>Area under the curve of the cumulative SUV-volume histogram (AUC_{CSH}),<br>Skewedness (S), Coefficient of variance (CoV), Kurtosis (K),<br>Integral Uniformity (IU), Differential Uniformity (DU),<br>Energy (E_H) and Entropy (Ent_H)
GLCM	Local Homogeneity (LH), Correlation (C_{CM}), Contrast (Con_{CM}),<br>Energy (E_{CM}), Entropy (Ent_{CM}), Variance (Var_{CM}),<br>Autocorrelation (Acor) and Dissimilarity (D)
GLRLM	Short Run Emphasis (SRE), Long Run Emphasis (LRE),<br>Low Gray-Level Run Emphasis (LGRE), High Gray-Level Run Emphasis (HGRE)<br>Short Run Low Gray-Level Emphasis (SRLGE), Short Run High Gray-Level<br>Emphasis (SRHGE), Long Run Low Gray-Level Emphasis (LRLGE),<br>Long Run High Gray-Level Emphasis (LRHGE),<br>Gray-Level Non-uniformity (GLN), Run Percentage (RP)<br>Length Non-uniformity (RLN)
GLSZM	Small Zone Emphasis (SZE), Large Zone Emphasis (LZE),<br>Low Gray-Level Zone Emphasis (LGZE), High Gray-Level Zone Emphasis (HGZE),<br>Small Zone Low Gray-Level Emphasis (SZLGE), Small Zone High<br>Gray-Level Emphasis (SZHGE), Large Zone Low Gray-Level Emphasis (LZLGE),<br>Large Zone High Gray-Level Emphasis (LZHGE),<br>Zone-Size Non-uniformity (ZSN), Zone Percentage (ZP),<br>Gray-Level Variance (GLV) and Zone-Size Variance (ZSV)
NGTDM	Coarseness (Coar), Contrast (Con_{NM}),<br>Complexity (Comp) and Texture Strength (TS)

Abbreviations: SUV: standardized uptake values, GLCM: gray-level co-occurrence matrix, GLRLM: gray-level run length matrix, GLSZM: gray-level size zone matrix, NGTDM: neighborhood gray tone difference matrix.

Table S2. Radiomics features with prognostic value at W2 in the initial cohort.

OS	DICE, Energy, C_{CM}, LZE, LZLGE, WFGLV
PFS	DICE, C_{CM}, LZLGE, WF-C_{CM}, WF-GLV, WF-GLV2, WF-Busyness, Q-SZLGE,<br>Q-LGRE, QSRLGE
LR	LGZE, DU, SZLGE, SRLGE, LGRE, LRLGE<br>WF-SZLGE, WF-GLV2, Q-SZLGE, LZLGE, WF-LZHGE, WF-LGRE<br>WF-SRLGE, WF-LGZE, WF-LRLGE, Q-LGZE, C_{CM}<br>GLV2
DM	DICE, Q-C_{CM}

Abbreviations: OS: overall survival, PFS: progression free survival, LR: local recurrence and DM: distant metastasis.

**Outcome**					**W2**		**W5**		
**Patient**	**Locoregional recurrence**	**Distant metastasis free survival**	**Free Survival (months)**	**Overall Survival (months)**	**ΔV**	**CP (DICE for estable, Sens for increasing and PPV for deacreasing hypoxia)**	**ΔV**	**CP (DICE for estable, Sens for increasing and PPV for deacreasing hypoxia)**	**W2 vs W5**
**P01**			60	60	-1		236		
**P02**			59	59	281		319		
**P03**			61	61	-68		109		
**P04**	1		8	58	22		-100		
**P05**	1		11	55	16		-100		
**P06**	1	1	7	34	-92		-100		
**P07**			5	9	-100		-98		
**P08**			49	49	-82		-84		
**P09**		1	44	44	-93		-100		
**P10**	1	1	10	24	-70		-98		
**P11**	1		10	16	136		-74		
**P12**	1		8	12	-96		-100		
**P13**			41	41	-93		-100		
**P14**	1	1	7	30	0		0		
**P15**		1	3	16	-95		324		
**P16**	1		8	31	-99		-100		
**P17**	1		17	30	-78		-89		
**P18**			27	27	96		-40		
**P19**	1		14	28	-43		-86		
**P20**	1	1	10	18	-16		-100		
**P21**	1		8	17	-12		-68		
**P22**			23	24	-48		-58		
**P23**			18	19	41		-18		
**P24**			19	19	-90		-100		
**P25**			14	15	-97		-34		

	No Recurrence	No Metastasis	> median (14)	> median (28)
1	Recurrence	Metastasis	<=median (14)	<=median (28)

**Status based on ΔV**	
Increasing (IH):	ΔV ≥ 15%
Stable (SH):	15% > ΔV> -15%
Decreasing (DH):	-15% ≥ ΔV

**Status based on CP**	
Static, geographically Stable (gS):	CP≥0,6
Dynamic, geographically unestable (gD):	0,60>CP
Without hypoxic region	

Different Status
Same status

Figure S1. Hypoxia variation status and treatment outcome for each patient and in each week.
